# Functional connectome fingerprint of sleep quality in insomnia patients: Individualized out-of-sample prediction using machine learning

**DOI:** 10.1016/j.nicl.2020.102439

**Published:** 2020-09-18

**Authors:** Xiaofen Ma, Dongyan Wu, Yuanqi Mai, Guang Xu, Junzhang Tian, Guihua Jiang

**Affiliations:** a Department of Medical Imaging, Guangdong Second Provincial General Hospital, Guangzhou, PR China; b Department of Neurology, China-Japan Friendship Hospital, Beijing, PR China; c Department of Radiology at Maoming General Hospital, Maoming, PR China; d Department of Neurology, Guangdong Second Provincial General Hospital, PR China

**Keywords:** Insomnia disorder, Pittsburgh sleep quality index (PSQI), Individualized out-of-sample prediction, Machine learning, Functional connectivity

## Abstract

**Objectives:**

Insomnia disorder has been reclassified into short-term/acute and chronic subtypes based on recent etiological advances. However, understanding the similarities and differences in the neural mechanisms underlying the two subtypes and accurately predicting the sleep quality remain challenging.

**Methods:**

*Using 29 short-term/acute insomnia participants and 44 chronic insomnia participants*, we used whole-brain regional functional connectivity strength to predict unseen individuals’ Pittsburgh sleep quality index (PSQI), applying the multivariate relevance vector regression method. Evaluated using both leave-one-out and 10-fold cross-validation, the pattern of whole-brain regional functional connectivity strength significantly predicted an unseen individual’s PSQI in both datasets.

**Results:**

There were both similarities and differences in the regions that contributed the most to PSQI prediction between the two groups. Further functional connectivity analysis suggested that between-network connectivity was re-organized between short-term/acute insomnia and chronic insomnia.

**Conclusions:**

The present study may have clinical value by informing the prediction of sleep quality and providing novel insights into the neural basis underlying the heterogeneity of insomnia.

## Highlight

• Short-term and chronic insomnia are two subtypes of insomnia.
• Functional connectome predicts individual sleep quality for both two subtypes.
• Shared and distinct neural basis underlying poor sleep quality between two subtypes.

## 1 Introduction

Insomnia disorder is the second-most common mental disorder, characterized by frequent or constant difficulty in falling asleep, poor sleep maintenance, and inadequate sleep satisfaction (Morin et al., 2015). According to one epidemiological report (Ohayon, 2002), around 10% of adults suffer from this condition. Insomnia disorder severely affects quality of life and has bidirectional association with various medical, neurological, and mental disorders. As such, it greatly increases healthcare consumption, work disability, and costs to society (Palagini et al., 2016). However, the underlying pathophysiology of insomnia disorder is poorly understood, and accurate prediction of sleep quality of insomnic patients remains challenging.

Using resting state functional magnetic resonance imaging (rs-fMRI), prior studies have consistently demonstrated abnormal spontaneous regional brain activity in patients with insomnia disorder. For example, these patients showed lower spontaneous activity in regions of higher-order cognitive networks (Li et al., 2016) and higher activity in sensory/perception-related regions (Zhou et al., 2017). Considering the interaction between regions, several studies have demonstrated abnormalities in both local regional homogeneity (Wang et al., 2016) and distributed functional connectivity among regions spanning the frontal, subcortical, and parietal cortex in patients with insomnia disorder (O'Byrne et al., 2014, Bagherzadeh-Azbari et al., 2019). All these studies sought to infer patterns of abnormal brain functional activity that are common across patients. However, each patient with insomnia is a unique case, and these studies ignored the considerable heterogeneity among patients with insomnia (Finn et al., 2015, Rosenberg et al., 2015, Gabrieli et al., 2015).

Several recent studies have explored the underlying neural basis of individual differences in insomnia disorder. Li et al. (Li et al., 2016) and Zhou et al. (Zhou et al., 2017) found that the amplitude of low frequency fluctuations was related to the Pittsburgh sleep quality index (PSQI) in both the inferior parietal lobule and postcentral gyrus. At the functional connectivity level, the decreased overall connectivity between the left inferior frontal gyrus and the rest of the brain was related to low PSQI (Yan et al., 2018), and the connectivity between inferior parietal lobule and striatum was positively correlated with PSQI (Wang et al., 2018) across the population with insomnia. However, these studies relied on in-sample correlation inference, so it is unknown if the observed correlation could be generalized to unseen individuals (Gabrieli et al., 2015). As such, they possessed little clinical value. Moreover, these studies used mass-univariate analysis and ignored the relationship between the multivariate pattern of functional connectivity and sleep quality.

To address these problems, connectome-based individualized prediction methods were developed (Finn et al., 2015, Rosenberg et al., 2015, Cui and Gong, 2018, Cui et al., 2020) using cross-validation (CV) approaches, which inherently evaluate the model’s out-of-sample generalizability to unseen individuals (Gabrieli et al., 2015). Typically, a specific behaviour score is initially estimated in a connectivity-based predictive model using training samples; it is then validated using independent testing samples. This approach generally employs machine learning, which is a multivariate pattern analysis approach that can capture the relationship between the complex pattern of whole-brain features and behaviours, and can therefore provide more information beyond the traditional mass-univariate analysis. Once the model can generalize well within the testing samples, it captures the brain representation of the behaviour. This method has been applied to predict both cognitive performance and clinical symptoms, including intelligence quotient (Finn et al., 2015), attention ability (Rosenberg et al., 2015), language ability (Cui et al., 2018), and cocaine abstinence (Yip et al., 2019). However, it has not yet been used to predict sleep quality in patients with insomnia.

Thus, in the present study, we applied the multivariate relevance vector regression (RVR) method and whole-brain regional functional connectivity strength to predict unseen sleep quality in patients with insomnia. In particular, we focused on two patient populations: those with short-term/acute insomnia and those with chronic insomnia (Sateia, 2014). We were interested in both the common and distinct underlying neural substrates between these insomnia types. Both leave-one-out (LOO) and 10-fold CVs were used to evaluate the generalizability of the model. Finally, we characterized the connectivity pattern among regions that related to individual differences in sleep quality among patients with chronic insomnia.

## 2 Materials and methods

### 2.1 Participants

We have recruited 30 patients with short-term/acute insomnia and 46 patients with chronic insomnia from either the Department of Neurology at Guangdong Second Provincial General Hospital, Guangzhou, China or the Department of Neurology at Maoming General Hospital, Guangdong, China between April 2016 and April 2018. The diagnostic criteria for short-term/acute and chronic insomnia disorder was according to the Diagnostic and Statistical Manual of Mental Disorders, version 5 (DSM-V) and the International Classification of Sleep Disorders, Third Edition (ICSD-3), with complaints of difficulty falling asleep, maintaining sleep or early awakening for at least 3 months and three times per week (chronic)/ at least three times per week but<3 months (short-term/acute). Patients with insomnia disorder (short-term/acute and chronic) were excluded due to (1) insomnia disorder secondary to severe mental condition (e.g., depression, anxiety, and epilepsy), (2) other sleep disorders, (3) history of significant head trauma or loss of consciousness for > 30 min, (4) history of medication-based treatment for insomnia disorder, (5) history of alcohol abuse, drug abuse, or smoking, (6) abnormal signal in conventional MRI imaging, (7) pregnancy, lactation, or menstruation, and (8) Hamilton Anxiety Scale (HAMA) score > 7 or Hamilton Depression Scale (HAMD) score > 7.

Finally, one short-term/acute insomnia group (N = 30) and one chronic insomnia group (N = 46) were included. The two datasets were acquired using two different scanners. All participants were asked to complete the PSQI (Buysse et al., 1989), the Epworth Sleepiness Scale (ESS) (Doneh, 2015) and ISI (Bastien et al., 2001), the HAMA (Thompson, 2015), and the HAMD (Worboys, 2013) to evaluate their sleep situation and mental status. In addition, all participants were right-handed, as assessed using the Edinburgh Handedness Inventory (Oldfield, 1971). The study was approved by the Ethics Committee of Guangdong Second Provincial General Hospital. All participants completed informed written consent before inclusion in the study.

### 2.2 Image acquisition

For both datasets, T1-weighted and rs-fMRI datasets was acquired. Furthermore, T2-FLAIR images were obtained for every participant to detect clinically silent lesions. Subjects were instructed to keep their eyes closed, stay awake, and remain still during rs-fMRI scanning. After scanning, all subjects confirmed they were awake during the scanning.

#### 2.2.1 Short-term/acute insomnia

The short-term/acute insomnia patients were scanned using a 3.0-T MR scanner (Skyra; Siemens, Germany) at the Department of Radiology, Maoming General Hospital. The rs-fMRI data were acquired using the following parameters: repetition time (TR) = 2000 ms, echo time (TE) = 30 ms, flip angle = 90°, slice thickness = 3.6 mm (with a 0.7 mm gap), voxel size: 3.6 × 3.6 × 3.6 mm, matrix = 64 × 64, field of view (FOV) = 240 × 240 mm^2^; 35 transverse-planes parallel with the anterior commissure–posterior commissure line were imaged, with 240 dynamic scans, for a total of 8,400 images. Additionally, individual high-resolution anatomical images were acquired using a 3D magnetization-prepared, rapid-acquisition, gradient-echo (MPRAGE), T1-weighted sequence: 160 axial slices, TR = 10.4 ms, TE = 4.3 ms, flip angle = 15°, slice thickness = 1.0 mm, no gap, matrix = 256 × 256, FOV = 256 × 256 mm^2^.

#### 2.2.2 Chronic insomnia

The chronic insomnia dataset patients were scanned using a 3.0-T MR scanner (Ingenia; Philips, the Netherlands) at the Department of Medical Imaging, Guangdong Second Provincial General Hospital. The rs-fMRI data were acquired using the following parameters: TR = 2000 ms, TE = 50 ms, flip angle = 90°, slice thickness = 3.6 mm (with a 0.7 mm gap), voxel size: 3.6 × 3.6 × 3.6 mm, matrix = 64 × 64, field of view (FOV) = 230 × 230 mm^2^, 35 transverse planes parallel with the anterior commissure–posterior commissure line were imaged, with 240 dynamic scans, for a total of 8,400 images. Additionally, individual high-resolution anatomical images were acquired using T1-weighted, 3D MPRAGE: 160 axial slices, TR = 25 ms, TE = 4.1 ms, flip angle = 30°, slice thickness = 1.0 mm, no gap, matrix = 256 × 256, FOV = 230 × 230 mm^2^.

During rs-fMRI data acquisition, participants were asked to lie quietly in the scanner with their eyes closed and not think of anything specifically. The rs-fMRI scan lasted for 8 min, and a total of 240 volumes were obtained for each participant. After the examination, all participants were asked questions to verify the degree of their co-operation.

### 2.3 Image pre-processing

We used data processing and brain imaging analysis (Yan et al., 2016) to pre-process the rs-fMRI data. This processing procedure included the following steps: (1) removing the first 10 functional volumes, (2) correcting for acquisition time delay between slices, (3) realigning all volumes to a selected reference volume to correct for head motion, (4) co-registering individual T1-weighted images to mean functional images, (5) segmenting the co-registered T1 images into grey matter, white matter, and cerebrospinal fluid tissue maps using Diffeomorphic Anatomical Registrations Through Exponentiated Lie Algebra (DARTEL) segmentation (Ashburner, 2007), (6) using the acquired transformation parameters to normalize the functional image to the Montreal Neurological Institute (MNI) space, and then re-sampling the image into 3-mm isotropic voxels, (7) removing the linear trend and several nuisance signals, including Friston’s 24 head motion parameters, global signal, and the average white matter and cerebrospinal fluid signals, (8) temporal bandpass filtering (0.01–0.1 Hz) was performed voxel-by-voxel.

One patient with short-term/acute insomnia was removed because of failure during normalization (See Supplementary Fig. 1). In the chronic insomnia group, one subject was removed due to head motion exceeding 3 mm and 3°during fMRI scanning, and another was removed because of motion artefact in the T1-weighted image (see Supplementary Fig. 2).

Ultimately, we included 29 subjects in the short-term/acute insomnia group and 44 subjects in the chronic insomnia group. See Table 1 for the demographic information of both datasets. *PSQI was not significantly correlated with age in either acute/short insomnia group (r = -0.13, p = 0.50) or chronic insomnia group (r = 0.006, p = 0.97). We calculated the mean root mean square (RMS) framewise displacement to measure the head motion for each subject. The head motion also did not significantly correlate with PSQI in either short insomnia group (r = -0.25, p = 0.19) or chronic insomnia group (r* = 0.08, *p* = 0.59*).*

**Table 1:** The demographic and clinical characteristics of insomnia participants (short-term/acute insomnia *N* = 29, Image acquisition by the Skyra; Siemens), (chronic insomnia *N* = 44, Image acquisition by the Ingenia; Philips,).

	Acute Insomnia (n = 29) Chronic Insomnia (n = 44)
Handedness(R/L)	29/0 44/0
Gender(M/F)	7/22 15/29
Age(years)	28.621 ± 6.961 38.068 ± 10.281
Education(years)	13.035 ± 3.581 10.159 ± 3.831
Smoking (Y/N)	0/29 0/44
Drinking(Y/N)	0/29 0/44
Course disease(weeks)	4.817 ± 4.052 65.955 ± 61.683
Drug treatment(Y/N)	0/30 0/44
PSQI	16.567 ± 3.159 18.432 ± 2.267
ISI	20.933 ± 6.236 20.136 ± 5.630
ESS	17.000 ± 4.871 9.046 ± 6.164

Values are represented as mean ± SD. R, right; L, left. M, male; F, female. Y, yes; N, no.

### 2.4 Whole-brain resting-state functional connectivity strength feature extraction

The human Brainnetome atlas (https://atlas.brainnetome.org/) was used, which parcellates the entire grey matter into 246 regions (123 in each hemisphere) consisting of 210 cortical and 36 subcortical regions (Fan et al., 2016). For each subject, a regional mean time series was calculated by averaging the time series over all voxels within the region, and thus a total of 246 regional mean time series were yielded. The resting-state functional connectivity (rsFC) between each pair of regions was computed using Pearson’s correlation between two regional mean time series. For each region, the nodal rsFC strength (rsFCS) was calculated, which corresponds to the centrality measure in graph theory and is simply defined as the sum of the rsFC values between that region and all other regions (245 in total) (Buckner et al., 2009, Liu et al., 2017). A whole-brain nodal rsFCS feature vector, with 246 features in total, was extracted for each subject. It was then used to predict behaviour in the subsequent analysis.

### 2.5 Individualized prediction of PSQI using nodal rsFCS

**Fig. 1:**
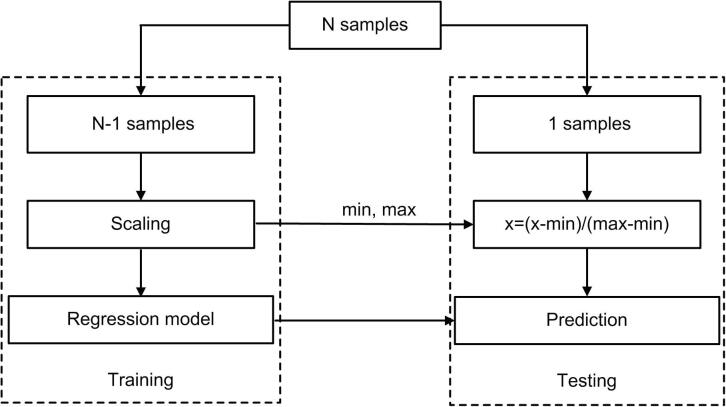
Schematic overview of one loop of leave-one-out cross-validation (LOOCV) prediction framework. One subject was used as testing and the remaining subjects were used as training dataset. Each feature was linearly scaled between zero and one across the training dataset, and the scaling parameters were also applied to scale the testing dataset. Relevance vector regression was used to train a model, which was used to predict the PSQI of the testing subject.

Based on whole-brain nodal rsFCS features, we applied multivariate RVR to predict individual differences in PSQI scores (Fig. 1). All codes are publicly released on Github (https://github.com/ZaixuCui/Pattern_Regression_Clean). RVR is formulated in a probabilistic Bayesian learning framework and obtains sparse solutions to a multivariate regression model (Tipping, 2001). The function takes the form as below:

$$f\left(x_{i}\right)=\sum_{s=1}^{l}\beta_{s}\left(x_{i}*x_{s}\right)+\beta_{0}$$

where *x_i_* is a high-dimensional feature vector *(x_i,1_, …, x_i,p_)* for the *i^th^* subject, *p* is the number of features, and $\beta_{s}$ is the regression coefficient of the *s^th^* feature. The samples (*l* < N), termed the ‘relevance vector’, are used to fit the model in RVR. An explicit zero-mean Gaussian prior was applied on the parameter β,

$$p\left(\beta |\alpha \right)=\prod_{i=0}^{N}ℵ\left(\beta_{i}|0,\alpha_{i}^{-1}\right)$$

and the most weights were set as zero. The samples (number *l* < N) with non-zero weights, termed the ‘relevance vector’, were used to train the model. Maximum likelihood estimation was used to find the weights of these samples. The regression coefficients of all features were determined as the weighted sum of the feature vector of all ‘relevance vector’ samples. This algorithm (Cui and Gong, 2018) has no algorithm-specific free parameter and is computationally more efficient than other algorithms. RVR has been widely used to predict age and behaviour. We used the codes from the PRoNTo toolbox (http://www.mlnl.cs.ucl.ac.uk/pronto/; Schrouff et al. (2013)) to implement RVR.

#### 2.5.1 Prediction framework

We applied LOOCV to estimate the generalizability of the model. Specifically, N-1 subjects were used as the training set and the remaining subjects were used as the testing set. Each feature was linearly scaled from a range of zero to one across the training dataset, and the scaling parameters were applied to scale the testing dataset (Cui and Gong, 2018, Cui et al., 2018). A prediction model was constructed using all the training samples and then used to predict the PSQI score of the testing sample. The training and testing procedure were repeated *N* times so that each subject was used once as the testing set. The Pearson correlation *r* and mean absolute error (MAE) between the predicted and observed PSQI were used to quantify the prediction accuracy (Cui and Gong, 2018).

#### 2.5.2 Significance of prediction performance

The permutation test was applied to determine whether the obtained correlation *r* and MAE values were significantly better than expected by chance (Cui and Gong, 2018). Specifically, the above prediction procedure was re-applied 1,000 times. For each time, we permuted the PSQI scores across the training samples without replacement. The *P*-value of correlation *r* was calculated as the proportion of a permutation that showed a higher value than that acquired in the real sample. The *P*-value of the MAE was the proportion of permutations that showed a lower value than that acquired in the real sample.

#### 2.5.3 Contributing regions

If the above prediction was significantly higher than that acquired by chance, the model had shown that the distributed representation in the brain was related to PSQI. We used all subjects to construct a new model that could identify the contributing regions to the model (Cui and Gong, 2018, Cui et al., 2018). The absolute value of the contribution weight represented the importance of the corresponding feature in the prediction (Cui and Gong, 2018, Cui et al., 2016). We defined the top 50 regions with the highest absolute contribution weight as the most contributing regions.

#### 2.5.4 Validation

We next conducted two additional analyses to validate our results. Firstly, we tested whether the predicted PSQI score was significantly correlated with observed PSQI score after controlling for ESS or head motion. Secondly, we applied 10-fold CV to validate the results acquired using LOOCV. We randomly split the data into 10 subsets, of which nine were used as training data and the remaining one was used as a testing set. We scaled the features on training data and then applied the acquired parameter to scale the testing data. We trained a prediction model using the training data, which was used to predict the PSQI of the testing data. This procedure was repeated 10 times, so that each subset was used as testing data once. As the split into 10 subsets was random, we repeated the above 10-fold CV procedure 20 times and reported the average prediction accuracy. Permutation testing (i.e., 1000 times) was used to evaluate the significance of the prediction accuracy.

Finally, for both the two groups, we evaluated the correlation between nodal strength and age controlling for sex, education for each of the 50 regions with the highest absolute contribution weight. FDR correction was used to account for multiple correction comparison. We also evaluated the correlation between nodal strength and ESS, controlling for age, sex and education, and the correlation between nodal strength and motion, controlling for age, sex and education.

### 2.6 Individualized prediction of PSQI using rsFC among the top 50 most contributing regions

Having demonstrated that nodal rsFC predicted an unseen individual’s PSQI score and identified the most contributing regions, we next sought to understand how functional connectivity among these regions contributed to this prediction. Specifically, we extracted the rsFC among the top 50 most contributing regions, resulting in a feature vector of 1,225 features for each subject. The acquired rsFC features were applied to predict an unseen individual’s PSQI, which was evaluated using the above LOOCV prediction framework. The top 50 connections with the highest absolute contribution weight were displayed.

In particular, the analysis using functional connectivity between the most contributing regions to predict PSQI may have involved overfitting. However, the aim of this analysis was to further understand how connectivity among these regions contributed to the prediction of sleep quality rather than to increase prediction accuracy. Moreover, it was not statistically certain that the connectivity among the most contributing regions could predict the PSQI.

## 3 Results

### 3.1 Whole-brain nodal rsFC predicted an unseen individual’s PSQI score

**Fig. 2:**
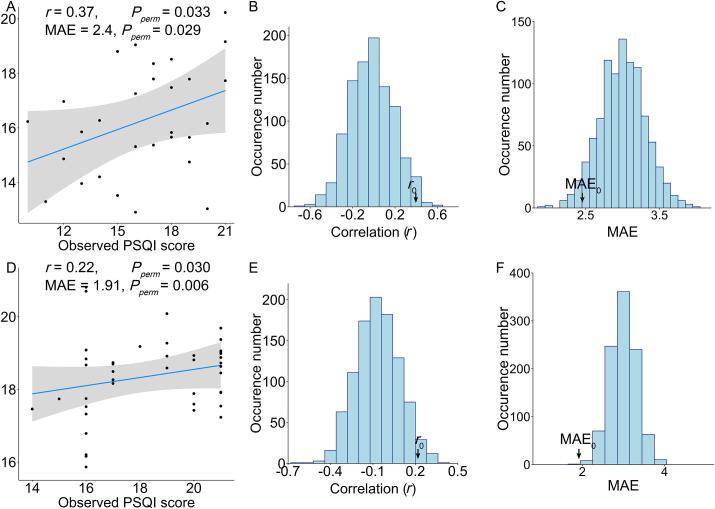
Whole-brain patterns of regional functional connectivity strength significantly predict an unseen individual’s sleep quality in both short-term/acute and chronic insomnia. (A) Scatter plot of the correlation between the observed and predicted PSQI scores across all patients with short-term/acute insomnia. The permutation distribution (1,000 times) suggests that both (B) the correlation r and (C) the mean absolute error (MAE) between the observed and predicted PSQI scores were significantly better than those acquired by chance in the short-term/acute insomnia group. Similarly, (D) for patients with chronic insomnia, both (E) the correlation r and (F) MAE between the observed and predicted PSQI scores are significantly better than those acquired by chance.

Evaluated using LOOCV, the partial Pearson’s correlation between observed and predicted PSQI scores was *r* = 0.37 controlling for age, sex, and education in patients with short-term/acute insomnia (Fig. 2**A**). A permutation test (1,000 permutations) suggested a significance of *P*_perm_ = 0.033 (Fig. 2**B**). The MAE between the observed and predicted PSQI scores was 2.4 (*P*_perm_ = 0.029) (Fig. 2**C**). In patients with chronic insomnia, the partial correlation *r* between observed and predicted PSQI scores was *r* = 0.22 (*P*_perm_ = 0.030) controlling for age, sex, and education, and the MAE was 1.91 (*P*_perm_ = 0.006) (Fig. 2**D, E, F**).

### 3.2 The most contributing regions for the prediction of PSQI score

**Fig. 3:**
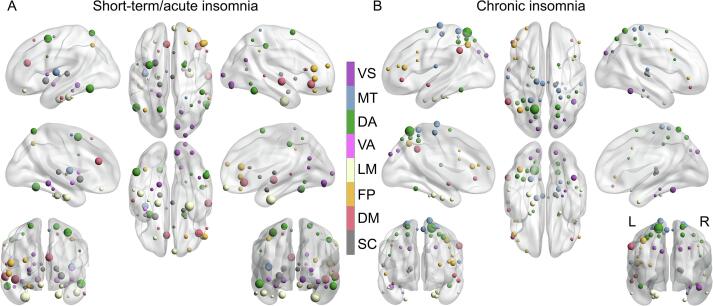
The regions with the highest absolute contribution weight in the PSQI prediction model in both (A) short-term/acute and (B) chronic insomnia groups. The 50 regions with the highest absolute contribution weight are displayed, with the colour representing the different cognitive systems. VS: visual; MT: motor; DA: dorsal attention; LM: limbic; FP: fronto-parietal; DM: default mode; SC: subcortical.

The regions contributing most to the prediction of PSQI were widespread, located in the parietal, temporal, and frontal areas in both the short-term/acute (Fig. 3**A, Supplementary** Table 1) and chronic (Fig. 3**B, Supplementary Table 2**) insomnia group. Some common regions, such as the pre-frontal and entorhinal cortex, parahippocampal gyrus, temporal gyrus, and thalamus, contributed greatly in both the short-term/acute and chronic insomnia groups. Some specific regions only contributed to one group but not the other. Specifically, the amygdala, insula, cingulate gyrus, and right frontal areas mainly contributed to PSQI prediction in the short-term/acute insomnia group (Fig. 3**A**), while the superior parietal lobule mainly contributed to PSQI prediction in the chronic insomnia group (Fig. 3**B**).

### 3.3 Validation analysis

First, after controlling for ESS and other covariates we used in the main analysis, the correlation between the predicted and observed PSQI scores was still significant in both short-term/acute insomnia group (*r* = 0.36, *P_perm_* = 0.037) and chronic insomnia group (*r* = 0.22, *P_perm_* = 0.04). Second, after controlling for head motion and other covariates we used in the main analysis, the correlation between the predicted and observed PSQI scores was still significant in and chronic insomnia group (r = 0.19, *Pperm* = 0.049) and had a trend to be significant in short-term/acute insomnia group (r = 0.30, *Pperm* = 0.057). Third, 10-fold CV suggested nodal strength significantly predicted PSQI scores (short-term/acute insomnia: *r* = 0.35, *P_perm_* < 0.001; MAE = 2.40, *P_perm_* < 0.001; chronic insomnia: *r* = 0.20, *P_perm_* < 0.001, MAE = 1.97, *P_perm_* < 0.001).

Finally, for each of regions contributing the most to the prediction, the nodal strength was not significantly correlated with age or ESS in either short-term/acute insomnia group or chronic insomnia group. Also, nodal strength was significantly correlated with head motion in only one brain region (i.e., dorsalmedial parietooccipital sulcus) in short-term/acute insomnia group and there is no significant correlation in chronic insomnia group.

### 3.4 Multivariate analysis revealed the relationship between rsFC and individual differences in PSQI score

**Fig. 4:**
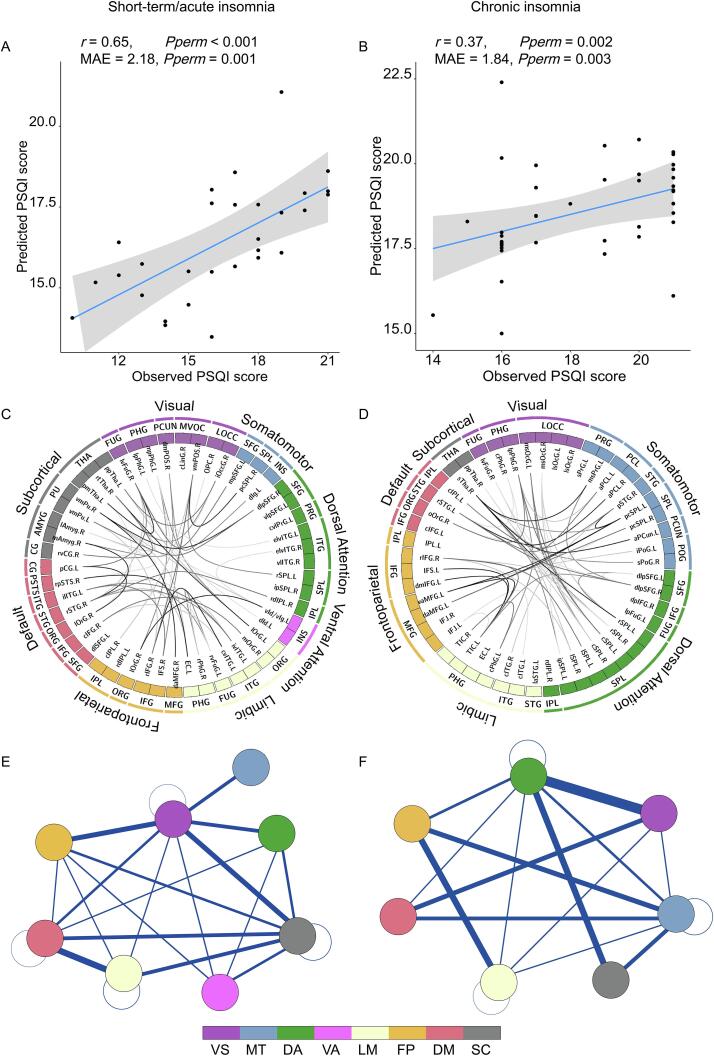
Multivariate predictive modelling further revealed the functional connectivity among the 50 most contributing regions that related to PSQI scores. The connectivity pattern among the 50 most contributed regions significantly predict the PSQI scores in both (A) the short-term/acute insomnia group and (B) chronic insomnia group. The between-region functional connectivity that contributed the most to PSQI prediction in both (C) the short-term/acute insomnia group and (D) the chronic insomnia group. The sum of the contribution weights of between-network connectivity in both (E) the short-term/acute insomnia group and (F) the chronic insomnia group. VS: visual; MT: motor; DA: dorsal attention; LM: limbic; FP: fronto-parietal; DM: default mode; SC: subcortical.

Having demonstrated that nodal rsFC predicted an unseen individual’s PSQI score and identified the regions that most contributed, we next sought to understand how functional connectivity among these regions contributed to the prediction. Using the rsFC of the top 50 most contributing regions to predict PSQI score, as evaluated using LOOCV, Pearson’s correlation between the observed and predicted PSQI scores was *r* = 0.65 (*P*_perm_ < 0.001) controlling for age, sex and education, and the MAE was 2.18 (*P*_perm_ = 0.001) in the short-term/acute insomnia group (Fig. 4**A**), while the correlation between the observed and predicted PSQI scores was *r* = 0.37 (*P*_perm_ = 0.002) controlling for age, sex, and education, and the MAE was 1.84 (*P*_perm_ = 0.003) in the chronic insomnia group (Fig. 4**B**).

Short-term/acute insomnia is deemed an early stage of chronic insomnia (Ellis et al., 2012). The functional connectivity that contributed most to PSQI prediction in short-term/acute insomnia included widespread functional connectivity among high-order cognitive systems (i.e., fronto-parietal, default mode network, etc.) (Fig. 4**C&E, Supplementary Table 3**). In contrast, in the chronic insomnia group, less functional connectivity between high-order cognitive systems contributed to the PSQI prediction (Fig. 4**D&F, Supplementary Table 4**). Notably, connectivity between the motor system and high-order cognitive systems did not contribute to PSQI prediction in the short-term/acute insomnia group, but contributed highly in the chronic insomnia group.

## 4 Discussion

Using two independent samples, we demonstrated that whole-brain nodal functional connectivity strength predicts unseen individuals’ sleep quality in both the short-term/acute and chronic insomnia groups. We found that some regions contributing most to the prediction of PSQI score in the short-term/acute and chronic groups were common to both groups, while others only contributed in one group. In particular, the emotional regulation neural circuit mainly contributed to prediction in the short-term/acute insomnia group, while the superior parietal areas mainly contributed to prediction in the chronic insomnia group. Further functional connectivity analysis suggested that between-network connectivity was re-organized during the cross-sectional transition from the short-term/acute insomnia stage to the chronic insomnia stage. Specifically, less between-network connectivity among high-order cognitive networks and more connectivity between the motor network and high-order networks contributed to PSQI prediction in the chronic insomnia group than in the short-term/acute insomnia group.

Prior studies relating insomnia to brain function have mainly focused on group comparison to investigate common abnormalities among patients with insomnia (Yan et al., 2018, O'Byrne et al., 2014). However, all individuals are unique in behavior, cognition, and brain function and structure. For example, Mueller and colleagues showed that there was a huge inter-subject variability in the functional connectivity, especially in the high-order association cortex (Mueller et al., 2013). Inter-subject variability in functional connectivity was related to the evolutionary cortical expansion and anatomical structure (i.e., sulcal depth), suggesting a potential evolutionary and anatomical root of inter-subject functional variability (Mueller et al., 2013). Moreover, it has been showed that genetic and environmental factors are critical in explaining the inter-individual variation in functional connectivity (Teeuw et al., 2019). All these factors make each individual unique. The individual uniqueness is the basis of individualized identification (Finn et al., 2015). Therefore, each patient with insomnia is also unique, and more recent studies have attempted to better understand the neural substrate underlying individual differences in insomnia.

Most existing studies of the individual differences in insomnia have used in-sample correlation to reveal the neural basis of individual differences (O'Byrne et al., 2014, Spiegelhalder et al., 2013), which has limited the generalizability of the findings. In contrast, the current study used the out-of-sample prediction method to demonstrate that whole-brain nodal functional connectivity strength predicted an unseen individual’s PSQI score. Specifically, the present study applied both LOO and 10-fold CVs to assess generalizability to unseen individuals, and significant prediction accuracies were achieved in both the short-term/acute and chronic insomnia groups. These models may possess clinical significance to predict sleep quality in patients with insomnia (Gabrieli et al., 2015).

More importantly, our study included two insomnia populations: one with short-term/acute insomnia and the other with chronic insomnia. The patients with short-term/acute insomnia had generally suffered from the condition for<3 months, while chronic insomnia was defined as a disease duration of > 3 months (Sateia, 2014). Recent evidence has demonstrated some symptom differences between short-term/acute and chronic insomnia. For example, patients with short-term/acute insomnia typically exhibit more life events, greater perceived stress, anxiety, and depression than normal sleepers, which corroborates Spielman’s model in which insomnia appears to be precipitated by stress (Spielman et al., 1987)^45^. In contrast, patients with chronic insomnia display signs of increased arousal, either on a cognitive-emotional, behavioural, autonomous, or central nervous system level (Riemann et al., 2015). Some literature has considered short-term/acute insomnia as an early stage of chronic insomnia (Riemann et al., 2017, Perlis et al., 2019). Short-term/acute insomnia can be relieved after cessation of the stressor because normal sleep shows plastic, automatic regulation. In contrast, chronic insomnia cannot, because other processes that interfere with sleep regulation are activated (Riemann et al., 2017, Espie, 2002). Individuals who have progressed from short-term/acute to chronic insomnia are more likely to develop first-onset depression (Ellis et al., 2014).

Some regions contributing most to the prediction of PSQI score in the short-term/acute and chronic groups were common to both groups, while others only contributed in one group. We observed that the pre-frontal areas, entorhinal cortex, parahippocampal gyrus, temporal gyrus, and thalamus contributed to PSQI prediction in both groups. These regions have been consistently found to be abnormal in insomnia patients compared to controls (O'Byrne et al., 2014, Zhou et al., 2017, Wang et al., 2018, Yan et al., 2018, Bagherzadeh-Azbari et al., 2019). The entorhinal cortex, parahippocampal gyrus, and temporal gyrus may play a crucial role in long-term memory encoding (Schon et al., 2016, Newmark et al., 2013, Park et al., 2011), while the pre-frontal area is critical for working memory (D'Esposito and Postle, 2015) and the thalamus is related to sleep regulation (Coulon et al., 2012). Behavioural studies have consistently suggested that patients with both short-term/acute and chronic insomnia suffer from sleep dissatisfaction and declines in memory consolidation (Cellini, 2017, Sutton, 2014). Moreover, this decline is positively related to sleep quality (Rana et al., 2018).

Emotion-related anterior meso-limbic regions, including the amygdala, insula, and cingulate gyrus, mainly contributed to PSQI prediction in the short-term/acute insomnia group, while the posterior-occipital areas, including superior parietal area, which is related to attention and spatial working memory (Jahn et al., 2012), mainly contributed to PSQI prediction in the chronic insomnia group. Prior studies have consistently suggested that acute stressors (e.g. stress at work, ill health, change in circumstances, or jet lag) usually trigger short-term/acute insomnia, while chronic insomnia can develop when short-term/acute insomnia occurs and becomes perpetuated through sleep-related cognition biases (Sutton, 2014, Riemann et al., 2017). Additionally, longitudinal studies have demonstrated that patients with acute insomnia often suffer stress-related emotional dysfunction or transient sleep disturbance behaviour (Yang et al., 2013), while patients with chronic insomnia tend to have selective impairments in spatial working memory or attention (Chen et al., 2016). Additionally, prior study demonstrated that chronic insomnia patients typically present higher levels of cyclic alternating pattern (CAP) fluctuation (Parrino et al., 2012), which present topographical location over the posterior parieto-occipital areas of the brain (Terzano and Parrino, 2000). This is consistent with our observation that the contributing regions of PSQI prediction in chronic insomnia mainly located in the posterior parieto-occipital areas. These results suggested a more emotionally-driven influence in the acute form of the insomnia versus a more introspective/self-mentation driven system in the chronic insomnia.

Furthermore, the results suggested that the specific functional connectivity related to sleep quality was re-organized during the cross-sectional transition from short-term/acute to chronic insomnia. In particular, in patients with short-term/acute insomnia, widespread between-network functional connectivity among high-order cognitive systems, such as fronto-parietal, default mode network, and subcortical systems contributed to PSQI prediction (Fig. 3**E**). In contrast, in patients with chronic insomnia, there were fewer connections among high-order cognitive systems that contributed to PSQI prediction (Fig. 3**F**). However, the connections between the motor system and other high-order cognitive systems did not contribute to PSQI prediction in short-term/acute insomnia (Fig. 3**E**) but contributed greatly to PSQI prediction in chronic insomnia (Fig. 3**F**). These results suggested that, in the early stages of insomnia (i.e., short-term/acute), there are perturbations in high-order cognition, whereas in later-stage insomnia (i.e., chronic), there perturbations in basic somatomotor functions. Consistent with this, prior literature has demonstrated that patients with chronic insomnia exhibit sensorimotor hyperarousal (Riemann et al., 2015). For example, patients with chronic insomnia generally show cortisol overproduction in the hypothalamic–pituitaryadrenal axis and activity in the autonomic nervous system (Riemann et al., 2015). In contrast, short-term/acute insomnia is a common experience for most people who experience stress; stress-related transient insomnia may further elicit maladaptive variations in cognitive behaviour and emotional arousal (Yang et al., 2013, Morin et al., 2015).

Several limitations of the current study should be addressed. First, the study was carried out in a small cohort; to generalize the results, further validation will be necessary using a large dataset of patients with insomnia. However, although we only used a small sample, our work used two independent samples from two scanners, which suggests that our results are robust to some extent. Second, our work used a cross-sectional sample; future studies should use a longitudinal design to explicitly examine the functional connectivity changes underlying the transition from the short-term/acute insomnia to chronic insomnia. Third, further studies could combine multiple neuroimaging features from different imaging modalities, such as grey matter volume, white matter microstructure integrity and cerebral blood flow, for better prediction of sleep quality of insomnic patients. Also, as prior work demonstrated (Cui and Gong, 2018), the prediction accuracy could be increased by training the model using more subjects. Forth, it should be noted that the acute and chronic groups were imaged on two different scanners. Although the same scanning parameters were used for rs-fMRI scanning, the scanner effect could impact the differences between the two groups. Fifth, as the attention during the rs-fMRI scan wasn’t tracked, so further studies may test if our observed differences between short-term/acute and chronic insomnia are related to the differences of attention during the scan (Laufs et al., 2003, Duyn, 2011).

## 5 Conclusions

In conclusion, the present study demonstrated the nodal functional connectivity strength predicted unseen individuals’ sleep quality in both short-term/acute and chronic insomnia. We further revealed changes in the functional connectivity pattern during the transition from the short-term/acute insomnia to chronic insomnia. The study may have clinical value by informing the diagnosis of sleep quality of insomnic patients, and may provide novel insights into the neural basis underlying the heterogeneity of insomnia. Finally, the present work showed that it is important to differentiate the stages of sleep quality in future studies.

## CRediT authorship contribution statement

**Xiaofen Ma:** Investigation, Writing - original draft, Funding acquisition. **Dongyan Wu:** Data curation, Methodology, Software, Visualization. **Yuanqi Mai:** Investigation, Software, Methodology. **Guang Xu:** Conceptualization, Investigation. **Junzhang Tian:** Funding acquisition, Writing - review & editing, Supervision. **Guihua Jiang:** Funding acquisition, Writing - review & editing, Project administration.

## Declaration of Competing Interest

The authors declare that they have no known competing financial interests or personal relationships that could have appeared to influence the work reported in this paper.
