# Supplementary material for: Functional connectome fingerprint of sleep quality in insomnia patients: Individualized out-of-sample prediction using machine learning
Source: Neuroimage Clin. 2020 Sep 18;28:102439. doi: 10.1016/j.nicl.2020.102439 (PMC7522804; doi:10.1016/j.nicl.2020.102439)
Supplement: Supplementary data 1 [file mmc1.docx]

**Supplementary Figure**


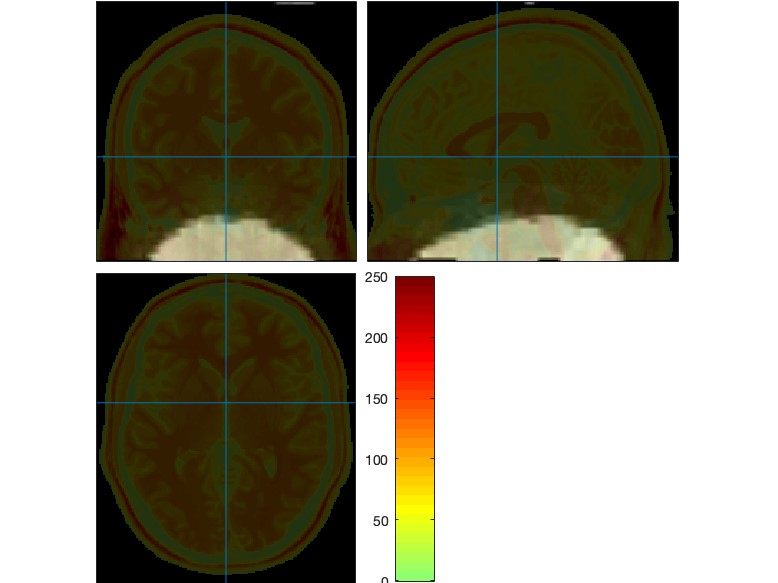


**Figure S1.** The subject in short-term/acute insomnia group with failure in normalization.


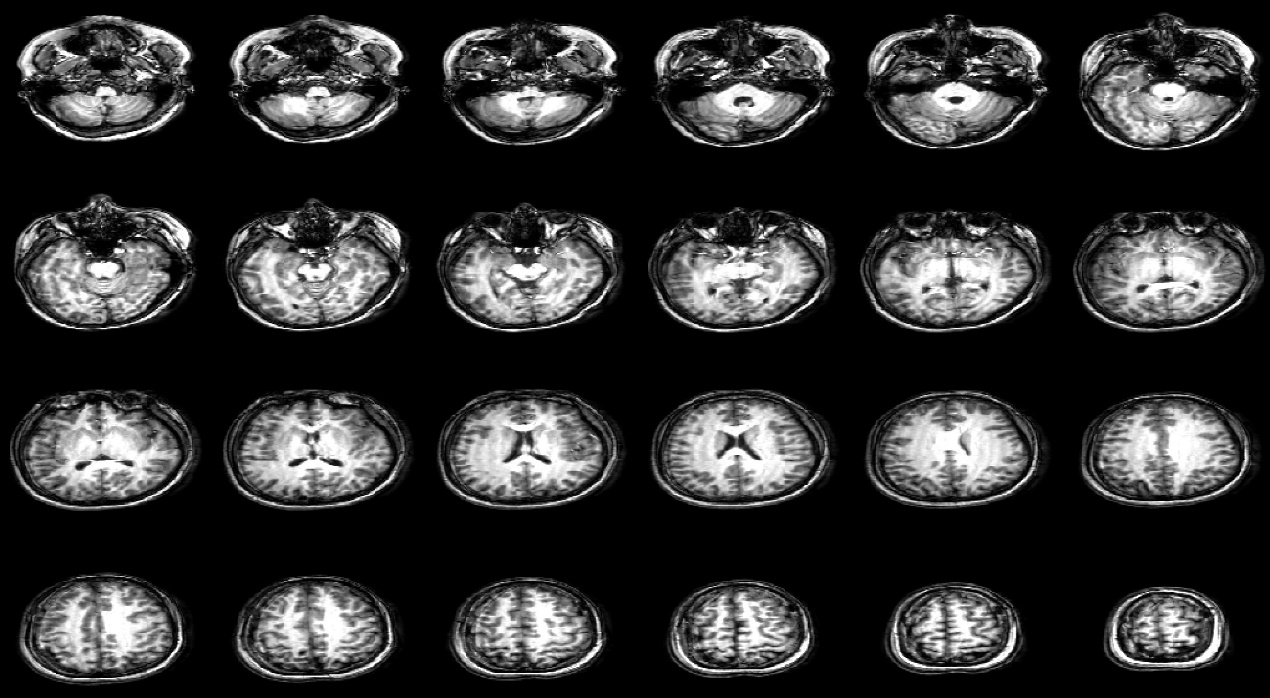


**Figure S2.** The T1-weighted image of the subjects with excess head motion.

**Table S1.** Top 50 regions that contributed the most to the prediction of PSQI in short-term/acute insomnia group.

**Table S2.** Top 50 regions that contributed the most to the prediction of PSQI in chronic insomnia group.

**Table S3.** Top 50 connections that contributed the most to the prediction of PSQI in short-term/acute insomnia group.

**Table S4.** Top 50 connections that contributed the most to the prediction of PSQI in chronic insomnia group.
